# Effectiveness of Steroid Treatment for SARS-CoV-2 Pneumonia With Cryptogenic Organizing Pneumonia-Like Reaction: A Case Report

**DOI:** 10.1017/dmp.2020.415

**Published:** 2020-10-26

**Authors:** Taehwa Kim, Eunjeong Son, Doosoo Jeon, Su Jin Lee, Seungjin Lim, Woo Hyun Cho

**Affiliations:** 1 Division of Pulmonology, Allergy and Critical Care Medicine, Department of Internal Medicine, Pusan National University Yangsan Hospital, Yangsan, Korea; 2 Division of Infectious Disease, Department of Internal Medicine, Pusan National University Yangsan Hospital, Yangsan, Korea

**Keywords:** COVID-19, cryptogenic organizing pneumonia SARS-CoV-2 pneumonia, steroid

## Abstract

Several studies on the treatment of coronavirus disease 2019 (COVID-19) are being conducted, and various drugs are being tried; however, the results have not been uniform. Steroids have been widely used in the treatment of COVID-19, but their effects are controversial. As immunosuppressive and anti-inflammatory agents, steroids are considered to reduce lung damage by regulating various inflammatory responses. We report a case of severe acute respiratory syndrome coronavirus-2 pneumonia manifesting as a cryptogenic organizing pneumonia-like reaction and discuss its treatment, clinical course, and favorable outcomes after steroid administration.

The role of steroids is important in decreasing the 28-d mortality rate associated with coronavirus disease 2019 (COVID-19), which requires oxygen supply or mechanical ventilation.^1^ Due to the negative outcomes reported by previous studies assessing steroid use for viral pneumonia, steroids have been considered controversial for treating certain viral pneumonias, such as Middle East respiratory syndrome and severe acute respiratory syndrome.^2,3^ The Surviving Sepsis Campaign guidelines recommend steroid treatment only for severe acute respiratory distress syndrome (ARDS) or refractory shock during COVID-19 treatment.^4^

However, in fact, steroid treatment has been widely used in many patients with COVID-19 pneumonia, its efficacy for this disease has been inconsistent.^1^ Severe acute respiratory syndrome coronavirus-2 (SARS-CoV-2) infection is characterized radiographically by extensive consolidation and ground glass opacities (GGOs), which indicate inflammatory manifestations^5^ causing diffuse lung damage and progression to pneumonia. The role of SARS-CoV-2 infection in diffuse lung injury is unclear, but it is expected that SARS-CoV-2 might damage the lungs by inducing immune responses. Steroids, as immunosuppressive and anti-inflammatory agents, can reduce lung damage by regulating various inflammatory responses.^6^ However, the issues of how and when to apply steroids remain unresolved, and it is necessary to investigate the clinical effects of steroids in patients with COVID-19 pneumonia.

Here, we report a case of COVID-19 pneumonia manifesting as a cryptogenic organizing pneumonia (COP)-like reaction and discuss its treatment, clinical course, and favorable outcome after steroid administration.

## Case Presentation

On February 27, 2020, a 71-y-old man presented with a purulent cough, mucoid sputum, chills, and sore throat but no fever. Two days after the onset of his initial symptoms, he developed fever (body temperature 38.0°C). He visited a public health center in Pusan on February 29, where a SARS-CoV-2 reverse transcription-polymerase chain reaction (RT-PCR) test was conducted at 8:00 pm. He tested positive for SARS-CoV-2 on March 1, and he was admitted to Gimcheon Medical Center and placed in a negative pressure room[Fig f1].

**Figure 1. f1:**
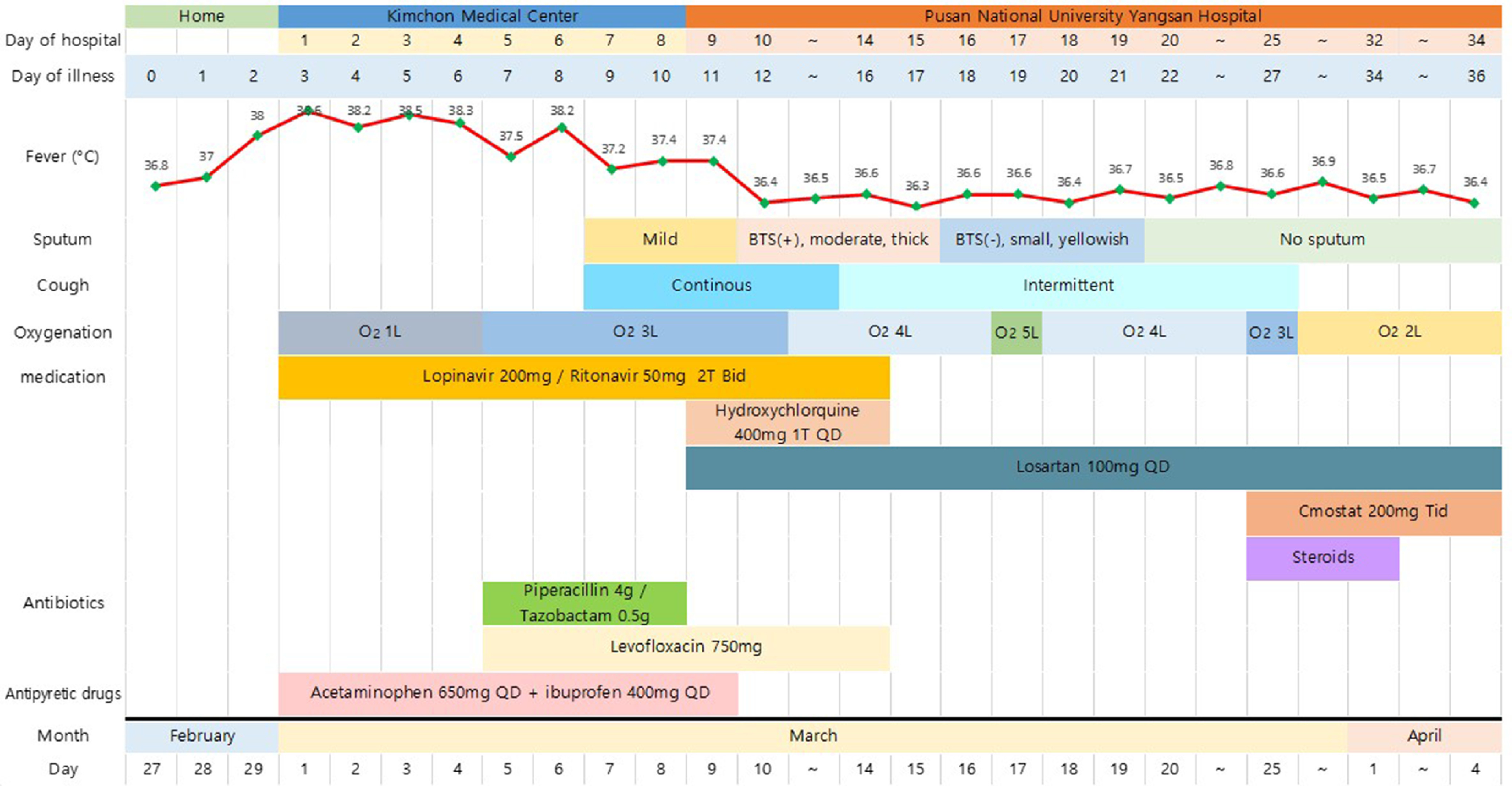
Clinical symptoms, fever, and treatment according to the day of illness and day of hospitalization.

The patient was otherwise healthy and did not take any medications. His height and weight were 173 cm and 83 kg, respectively. He was an ex-smoker (1 pack a day for 30 y) and had quit smoking in 2000. On the day of admission, the patient had uncontrolled fever with chills and myalgia. The physical examination results were normal, and there were no infiltrates on the chest radiograph (CXR). After admission, 2 tablets of lopinavir (200 mg)/ritonavir (50 mg) (Kaletra) were administered orally twice a day along with oxygen through a nasal cannula at 1 L/min. Additionally, supportive care, including rehydration with intravenous fluids and antipyretic drugs, was provided to control the symptoms.

On hospitalization day 4 (illness day 6), the patient’s condition worsened, and the oxygen levels had to be increased. Antibiotic treatment was started, specifically piperacillin (4 g)/tazobactam (0.5 g) intravenously every 8 h and levofloxacin (750 mg) intravenously every 24 h. On March 9 (illness day 11, hospitalization day 9), because his fever had persisted for 7 days and CXR findings had worsened, he was transferred to Pusan National Yangsan Hospital for intensive care[Table tbl1].

**Table 1. tbl1:** Results of RT-PCR tests for SARS-CoV-2 according to treatment progress

Specimen	Illness Day 11 March 9	Illness Day 15 March 13	Illness Day 18 March 16	Illness Day 19 March 17	Illness Day 28 March 6	Illness Day 35 April 3
Nasopharyngeal swab	Positive E gene Ct: 33.97 RdRp gene Ct: 34.66	Indeterminate E gene Ct: Not detected RdRp gene Ct: 35.30 N gene Ct: 36.72	Negative	Negative	Negative	Negative
Oropharyngeal swab	Positive E gene Ct: 34.82 RdRp gene Ct: 34.40	Indeterminate E gene Ct: Not detected RdRp gene Ct: Not detected N gene Ct: 38.85	Negative	Negative	Negative	Negative

Abbreviations: Ct, cycle threshold; RdRp, RNA-dependent RNA polymerase.

On the transfer day, the patient’s vital signs were as follows: pulse, 74 beats/min; respiratory rate, 20 breaths/min; blood pressure, 200/90 mmHg; and body temperature, 36.4°C. His oxygen saturation was 98% with nasal oxygen administered at 3 L/min. We continued oral administration of lopinavir (200 mg)/ritonavir (50 mg) twice a day and additionally administered losartan (100 mg) every 24 h and hydroxychloroquine (400 mg) every 24 h. Laboratory results were in the normal range, but CXR showed increased haziness and infiltrates in the right lower lobe (RLL). Furthermore, the patient reported mild amounts of yellowish sputum and intermittent cough and began producing blood-tinged sputum on day 12 of illness (hospitalization day 10).

The amount of sputum gradually increased and dyspnea symptoms worsened, causing the oxygen demand to increase continuously. Fortunately, the patient no longer had a fever. CXR showed pneumonia with increased infiltrates in the RLL compared with those on the initial CXR on hospitalization day 9. We maintained the oral administration of levofloxacin (750 mg) every 24 h. A week later, desaturation was observed even when the patient moved slightly. When he visited the bathroom, peripheral oxygen saturation dropped to as low as 86%. Oxygen was administered through a nasal cannula at 4 L/min. The pressure of oxygen was 63 mmHg in the arterial blood gas analysis (Figure 1)

RT-PCR tests were performed regularly, and the results were indeterminate on hospitalization day 13 (illness day 15) but finally became negative on hospitalization day 16 (illness day 18) (see Table 1).

**Figure 2. f2:**
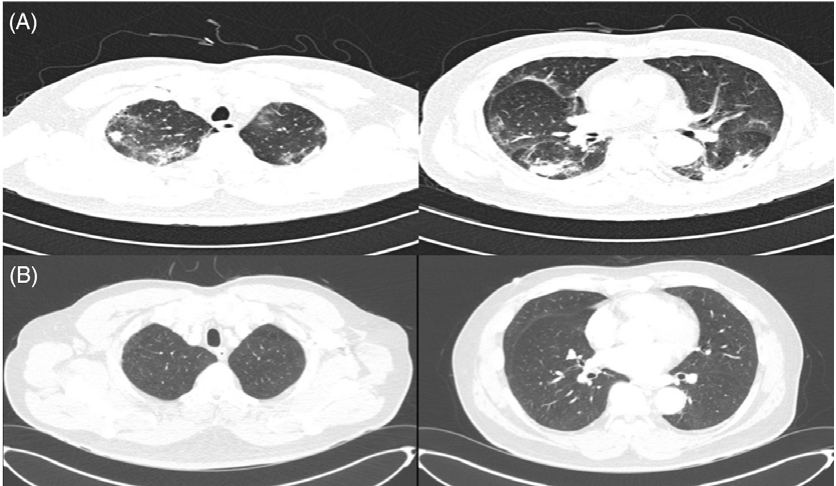
(A) Chest CT on illness day 20 and hospitalization day 18 (March 18). (B) Chest CT at the 3-Mo follow-up (July 4).

We decided to perform computed tomography (CT) of the chest because of the lack of change in CXR findings and dyspnea, despite supportive care[Fig f2]. Chest CT showed multiple patchy areas of consolidation, mainly in the sub-pleural zones of the RLL, and GGOs in both lungs, suggesting a COP-like reaction (see Figure 2A). We began steroid treatment on March 25 (illness day 27, hospitalization day 25). Dyspnea improved dramatically after 2 d, the oxygen demand decreased, and peripheral oxygen saturation consistently remained above 97%; oxygen was continually administered through a nasal cannula at 2 L/min. The patient was administered methylprednisolone (40 mg) from March 25 to 27, prednisolone (30 mg) from March 28 to 30, and prednisolone (20 mg) from March 31 to April 1. Following this, steroid treatment was discontinued, and continuous supportive care was provided. Chest CT at the 2-week follow-up showed a decreased extent of patchy consolidation and GGOs in both lungs, while irregular fibrotic lesions remained in the sub-pleural area of the RLL, indicating the interval resolving process of COP due to viral infection.

On April 9, all symptoms resolved, and he was finally discharged. After 3 mo, outpatient chest CT showed complete resolution of sub-pleural GGOs in both lungs (see Figure 2B).

## Discussion

The patient was treated for COVID-19 pneumonia manifesting as a COP-like reaction. Regardless of negative conversion on the SARS-CoV-2 RT-PCR test, there was no improvement in the patient’s symptoms of dyspnea and hypoxemia or radiologic findings. We performed chest CT for differential diagnosis, which revealed extensive GGOs, indicating a COP-like reaction. Despite conservative treatment for more than 20 d since the onset of symptoms, there was no effect of the treatment on COVID-19 pneumonia. These points indicated that the patient was probably having a COP-like reaction.

COP-like reactions can occur naturally in viral pneumonia, and the majority of cases resolve spontaneously.^7^ If CXR findings or symptoms aggravate, steroid treatment can produce a favorable result^8^; therefore, we decided to administer steroids to the patient, after which his symptoms improved rapidly. We tapered the dosage of steroids every 3 d, and the CXR findings showed continuous improvement. The patient was finally discharged, and complete resolution was confirmed on the follow-up chest CT after 3 mo.

Unlike common symptoms of infection during the early outbreak period, this case revealed that a patient with no improvement of imaging findings and continued hypoxia was suspected of having COP-like reaction based on the follow-up chest CT scan. He showed dramatic improvements after steroid administration. If symptoms persist even after the infection has been controlled following treatment for viral infection, it is reasonable to suspect a different diagnosis or a transition to another disease. Furthermore, considering the possibility that SARS-CoV-2 can cause a COP-like reaction can be helpful in planning treatment.

Recently, there have been many discussions on the effectiveness and application of steroid treatment in patients with COVID-19 pneumonia. For example, Horby and colleagues pointed out that steroid administration might positively decrease the 28-d mortality rate related to COVID-19.^1^ Moreover, they showed that steroids probably reduced the use of invasive ventilation. The World Health Organization recommends steroid therapy for patients with severe COVID-19.

Steroids are immunosuppressive and anti-inflammatory agents^6^ that are considered more effective in a state of progressive inflammatory response.^1^ In other words, the effectiveness of steroid treatment might be maximized after the inflammatory reaction is initiated. It is currently not known whether the effect of steroids shown in a recent study was due to the attenuation of the inflammatory response in ARDS^9^ or whether secondary viral organizing pneumonia^3^ induced improvement. However, this case suggests that, even if the effective mechanism is unclear, the potential of steroids for treating COVID-19 pneumonia with a COP-like reaction may be sufficiently confirmed.

Currently, there is no evidence of any associations among steroids, COVID-19 pneumonia, and secondary viral organizing pneumonia. Therefore, further research on the role of steroids in the treatment of COVID-19 pneumonia and secondary viral organizing pneumonia is required.
